# The satellite DNA AflaSAT-1 in the A and B chromosomes of the grasshopper *Abracris flavolineata*

**DOI:** 10.1186/s12863-017-0548-9

**Published:** 2017-08-29

**Authors:** Diogo Milani, Érica Ramos, Vilma Loreto, Dardo Andrea Martí, Adauto Lima Cardoso, Karen Cristiane Martinez de Moraes, Cesar Martins, Diogo Cavalcanti Cabral-de-Mello

**Affiliations:** 1 Departamento de Biologia, UNESP - Univ Estadual Paulista, Instituto de Biociências/IB, Rio Claro, São Paulo CEP 13506-900 Brazil; 2 Departamento de Morfologia, UNESP - Univ Estadual Paulista, Instituto de Biociências/IB, Botucatu, São Paulo Brazil; 3 Departamento de Genética, UFPE - Univ Federal de Pernambuco, Centro de Biociências/CB, Recife, Pernambuco Brazil; 4 IBS - UNaM – CONICET, Posadas, Misiones Argentina

**Keywords:** B chromosome, Repetitive DNA, Tandem repeat, Transcription

## Abstract

**Background:**

Satellite DNAs (satDNAs) are organized in repetitions directly contiguous to one another, forming long arrays and composing a large portion of eukaryote genomes. These sequences evolve according to the concerted evolution model, and homogenization of repeats is observed at the intragenomic level. Satellite DNAs are the primary component of heterochromatin, located primarily in centromeres and telomeres. Moreover, satDNA enrichment in specific chromosomes has been observed, such as in B chromosomes, that can provide clues about composition, origin and evolution of this chromosome. In this study, we isolated and characterized a satDNA in A and B chromosomes of *Abracris flavolineata* by integrating cytogenetic, molecular and genomics approaches at intra- and inter-population levels, with the aim to understand the evolution of satDNA and composition of B chromosomes.

**Results:**

AflaSAT-1 satDNA was shared with other species and in *A. flavolineata*, was associated with another satDNA, AflaSAT-2. Chromosomal mapping revealed centromeric blocks variable in size in almost all chromosomes (except pair 11) of A complement for both satDNAs, whereas for B chromosome, only a small centromeric signal occurred. In distinct populations, variable number of AflaSAT-1 chromosomal sites correlated with variability in copy number. Instead of such variability, low sequence diversity was observed in A complement, but monomers from B chromosome were more variable, presenting also exclusive mutations. AflaSAT-1 was transcribed in five tissues of adults in distinct life cycle phases.

**Conclusions:**

The sharing of AflaSAT-1 with other species is consistent with the library hypothesis and indicates common origin in a common ancestor; however, AflaSAT-1 was highly amplified in the genome of *A. flavolineata*. At the population level, homogenization of repeats in distinct populations was documented, but dynamic expansion or elimination of repeats was also observed. Concerning the B chromosome, our data provided new information on the composition in *A. flavolineata*. Together with previous results, the sequences of heterochromatic nature were not likely highly amplified in the entire B chromosome. Finally, the constitutive transcriptional activity suggests a possible unknown functional role, which should be further investigated.

**Electronic supplementary material:**

The online version of this article (10.1186/s12863-017-0548-9) contains supplementary material, which is available to authorized users.

## Background

For more than half a century, researchers have attempted to understand the complexity and evolution of the repetitive DNA fraction of the genome. Repetitive DNAs are classified depending on the rate of repetitiveness and arrangement in the genome and include Transposable Elements (TEs) and minisatellite, microsatellite and satellite DNA (satDNA) [1, 2]. The satDNAs are a highly repetitive fraction of the genome that are organized by repetitions directly contiguous to one another, in tandem, forming long arrays with hundreds of copies generally composed of 100–1000 base pairs [3]. These in tandem arrays compose most of the content of heterochromatin in eukaryotes, associated generally with the centromeres and telomeres [3–6]. These sequences evolve by the homogenization and fixation of different variants in a determined sexual population under the process of concerted evolution [7, 8]. Molecular mechanisms of DNA turnover, such as unequal crossing over and gene conversion, are primarily responsible for this homogenization pattern, leading to quantitative changes between species and generating specific satDNA subfamilies with differential arrangements and organization in a given genome [2, 9, 10]. For satDNA transcription, multiple functions are indicated to date [11], such as acting through RNAi as an epigenetic regulator of heterochromatin [12], playing a role as a structural element of centromeres, such as the alpha satellite in humans [13], and also regulating gene expression in yeast [14].

B chromosomes, which are extra elements to the standard complement (A complement) of some species, occur in approximately 15% of eukaryotes [15–20]. These chromosomes are known to be dispensable for normal development, for not recombining with standard A chromosomes and for their potential to present a drive mechanism and for accumulation [21]. Very characteristically, B chromosomes frequently accumulate repetitive DNAs, including satDNAs. Because of the repetitive DNA content, for a long time, B chromosomes were considered genetically inert [21]. However, studies revealed genes in B chromosomes [22–27] that could be transcriptionally active, such as in *Crepis capillaris* [28], *Capreolus pygargus* [29], *Eyprepocnemis plorans* [30], and *Trichogramma kaykai* [31]. Moreover, sequences in B chromosomes can influence expression patterns from genes in A chromosomes [32–34]. These data suggest a putative biological role for B chromosomes in some species.

Although frequently reported in some groups, information on satDNA populating B chromosomes in insects is restricted to a few species, such as the grasshoppers *Eyprepocnemis plorans* [15, 35, 36] and *Eumigus monticola* [37], wasps [38, 39], *Glossina* [40] and *Drosophila subsilvestris* [41]. In these B chromosomes, a direct association is observed between heterochromatin and accumulation of repetitive DNAs. In the organisms listed above, the isolated satDNAs helped in tracking the origin and evolution of B chromosomes.

In the grasshopper *Abracris flavolineata* with karyotype composed of 2n = 23, ×0 (males), one or two submetacentric B chromosomes were detected in individuals from a population sampled in Rio Claro/SP-Brazil [24]. Some studies address B chromosome origin and evolution in this species using repetitive DNAs as markers [24, 42, 43], revealing clues about B chromosome composition and origin. Here, we used restriction enzymatic digestion to isolate the first satDNA in the species, named AflaSAT-1, with the aim to further understand satDNAs among grasshoppers at the population level and their contribution to B chromosome structure and evolution in *A. flavolineata*. Finally, AflaSAT-1 was studied using a combination of chromosomal and molecular analyses at the population level to uncover the genome organization and evolution in A and B chromosomes.

## Results

### Isolation and characterization of satDNA sequences

**Fig. 1:**
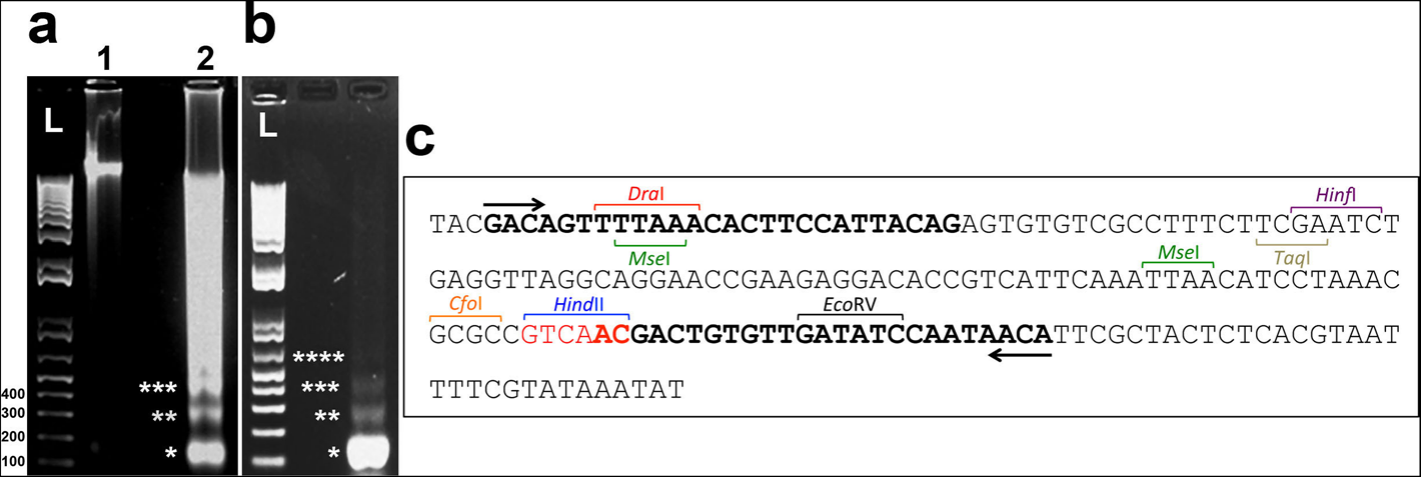
Agarose gel electrophoresis (**a**) of integrated genomic DNA (1) and the same sample digested with *Hind*II (2); (**b**) result of amplification of AflaSAT-1; (**c**) sequence of an entire monomeric unit of AflaSAT-1. In (**a**, **b**), L corresponds to 1 Kb plus marker, and asterisks indicate the monomer, dimer, trimer and tetramer. The arrows in (**c**) show the internal primers

The genomic DNA from individuals of the Rio Claro/SP population was digested using three different restriction endonucleases (RE), i.e., *Hind*II, *Alu*I and *Sma*I. A ladder pattern was revealed for *Hind*II and *Sma*I enzymes with bands of approximately 150, 300, and 450 bp, corresponding to the monomer and multimers of a putative tandem sequence. The monomer generated by *Hind*II was selected for further analysis (Fig. 1a). The monomer generated a sequence of 173 bp, after cloning and sequencing analysis. A convergent internal primer was designed and recovered a PCR fragment containing 137 bp, which formed a ladder pattern, typical for satDNAs (Fig. 1b, c). This sequence was named AflaSAT-1. Internal restriction sites for other enzymes were also recognized in AflaSAT-1 (Fig. 1c).

With PCR, AflaSAT-1 was detected in individuals in six populations that were sampled and in the μB-DNA from the Rio Claro/SP population. A total of 79 AflaSAT-1 sequences were recovered after cloning of individuals from Rio Claro/SP (15 clones), μB-DNA from Rio Claro/SP (10 clones), Cabo/PE (11 clones), Santa Bárbara do Pará/PA (10 clones), Paranaíta/MT (11 clones), Manaus/AM (12 clones), and Posadas/AR (10 clones). The AflaSAT-1 was similar in composition in all populations, with approximately 41.0% G + C base pairs. Different numbers of haplotypes were recognized depending on the population. Considering all sequences, 11 haplotypes were recognized. The number of mutations and variable sites was slightly higher in the sequences recovered from μB-DNA than in those from A chromosomes of the six populations. Nucleotide and haplotype diversity was also slightly variable, reaching the highest values in the Cabo/PE population (Table 1).

**Table 1:** Polymorphisms of AflaSAT-1 in distinct populations of *A. flavolineata* and from μB-DNA

	Rio Claro/SP 0B gDNA	Rio Claro/SP μB-DNA	Manaus/AM	Paranaíta/MT	Cabo/PE	Sta Bárbara do Pará/PA	Posadas/AR	All
Number of sequences	15	10	12	11	11	10	10	79
Number of haplotypes	4	3	5	3	5	3	4	11
Number of sites	137	137	137	137	137	137	137	137
Number of variable sites	4	7	5	3	5	3	4	10
Number of mutations	4	6	5	3	5	3	4	10
G + C proportion	44.5%	44.6%	44.3%	44.3%	45.0%	44.5%	44.5%	44.5%
Nucleotide diversity (π)	0.00876	0.01217	0.01615	0.01035	0.01859	0.01071	0.01184	0.01951
Standard deviation (π)	0.00204	0.00725	0.00211	0.00172	0.00187	0.00165	0.00255	0.00141
Haplotypes diversity (Hd)	0.600	0.511	0.833	0.655	0.855	0.689	0.644	0.845

**Fig. 2:**
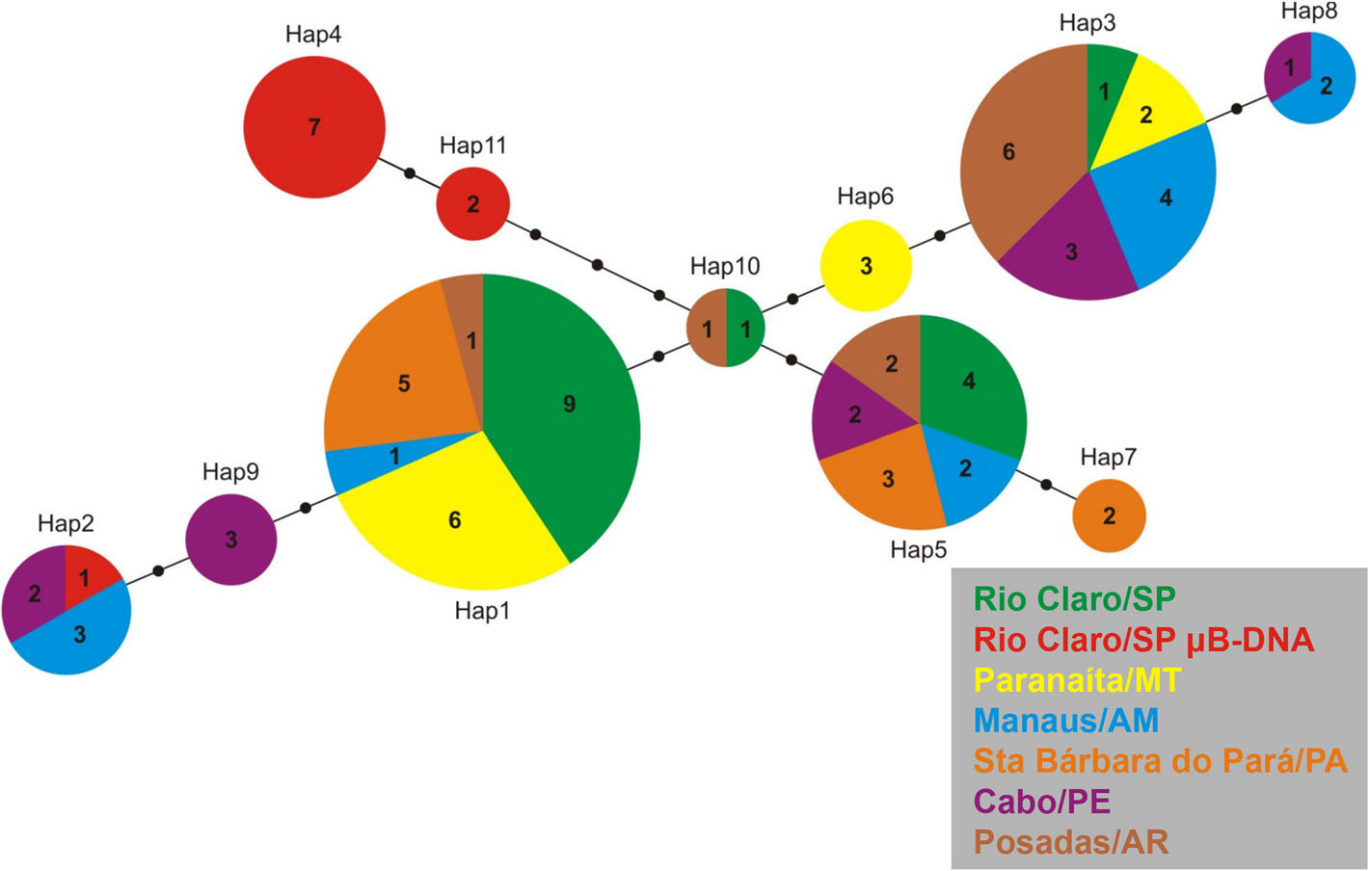
Haplotype network for the 11 haplotypes and their relationships from sequences of AflaSAT-1 obtained from different populations. Black dots correspond to substitutions, and the haplotype circle diameter corresponds to abundance. The different populations are represented in different colors

A minimum spanning tree revealed the relationships among the 11 haplotypes from all populations (Fig. 2). The tree of the 11 haplotypes was shared among at least five populations (Hap1, Hap3 and Hap5). The difference among the haplotypes was only one mutation (substitution), except for the haplotypes from exclusive μB-DNA that were differentiated by three or four mutations, Hap4 and Hap11. Finally, the μB-DNA presented a sequence grouped to Hap2, which occurred in Cabo/PE and Manaus/AM populations (Fig. 2).

Using the sequenced genome from one individual harboring one B chromosome, we identified one cluster that was similar to AflaSAT-1 in the assembly of RepeatExplorer. This cluster represented 1.04% of the genome, corresponding to the fourth most representative sequence. The cluster retrieved a total of 32 contigs, but only 19 entire sequences of AflaSAT-1 could be recovered, in addition to truncated sequences. Unexpectedly, in a few contigs and occurring with AflaSAT-1, a different sequence containing 242 bp was observed, which was named AflaSAT-2. For this repeat, we recovered ten entire sequences and some truncated repeats. Two arrangements were observed in the recovered contigs, only the AflaSAT-1 and AflaSAT-1 plus AflaSAT-2; the AflaSAT-2 was never recovered alone.

### Chromosome distribution and copy number variation of AflaSAT-1 in populations of *A. flavolineata*

**Fig. 3:**
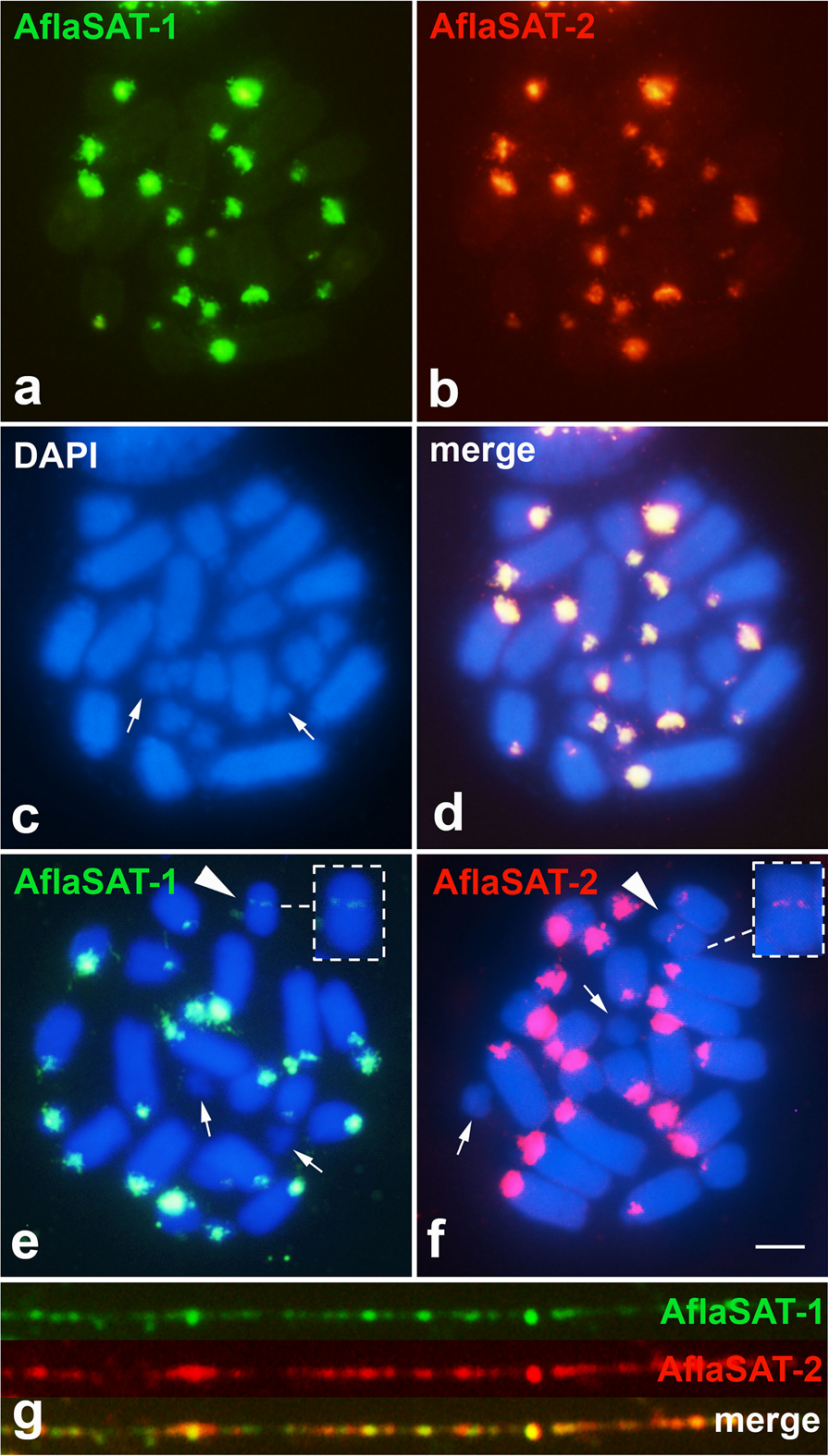
FISH in mitotic embryo chromosomes (**a**-**f**) and in distended chromatin fiber (**g**) of AflaSAT-1 and AflaSAT-2 satDNAs in individuals without (**a**-**d**, **g**) and with one B chromosome (**e**, **f**). Arrows in (**c**, **e**, **f**) show the pair 11 and the arrowhead in (**e**, **f**) the B chromosome. In (**e**, **f**), the B chromosome is highlighted in an inset. Each probe is indicated directly in the panel. Bar = 5 μm

The FISH using AflaSAT-1 and AflaSAT-2 probes in individuals from Rio Claro/SP revealed large pericentromeric blocks in all chromosomes, with blocks varying in size, except for pair 11 that did not have visible signals (Fig. 3a-d). The B chromosome showed a small signal in the centromeric region (Fig. 3e, f). These two sequences were interglimed with one another, as observed in fiber-FISH experiments (Fig. 3g). Association of AflaSAT-1 and AflaSAT-2 forming a composed unit was also observed in sequences retrieved from sequenced genomes.

**Fig. 4:**
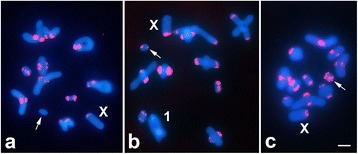
Chromosomal location of AflaSAT-1 in three different populations of *A. flavolineata*. (**a**) Sta Bárbara do Pará/PA, (**b**) Cabo/PE and (**c**) Posadas/AR. The X chromosome is indicated, and the arrows point to the pair 11. In (**b**), the largest chromosome, chromosome 1, is also indicated

Analysis of chromosomal distribution of AflaSAT-1 in different populations revealed identical patterns for individuals from Rio Claro/SP and from Sta. Bárbara do Pará/PA (Fig. 4a). In the other populations, a slight variation was observed, i.e., in Cabo/PE, the pair 1 did not have a signal and the pair 11 had a small positive signal (Fig. 4b), whereas individuals from Posadas/AR had signals in all chromosomes (Fig. 4c).

**Fig. 5:**
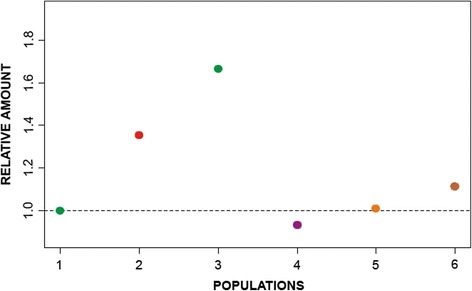
Relative quantification of AflaSAT-1 from different populations of *A. flavolineata* in comparison with Rio Claro/SP 0B male individuals. (1) Rio Claro/SP male 0B, (2) Rio Claro/SP male 1B, (3) Rio Claro/SP female 0B, (4) Cabo/PE male 0B, (5) Sta Bárbara do Pará/PA male 0B, (6) Posadas/AR male 0B

Additionally, we observed copy number variation of AflaSAT-1 with qPCR. Males without and with one B chromosome from Rio Claro/SP showed differences, with the individuals harboring the B chromosome having a higher copy number, as expected. Females from Rio Claro/SP also had a higher copy number, which was attributed to two copies of the X chromosome. Compared with those of Rio Claro/SP, individuals from Cabo/PE had fewer copies, whereas individuals from Santa Bárbara do Pará/PA had similar copy numbers. Finally, individuals from Posadas/AR had the highest number of copies (Fig. 5). The high standard deviations, considering the absolute copy number quantified, indicated that the copy number of AflaSAT-1 is very unstable and variable individually (Additional file 1).

### Occurrence of AflaSAT-1 in other grasshopper species

In the dot blot test for AflaSAT-1, among the six species tested, none revealed positive signals, and positive hybridization was restricted to genomic DNA from *A. flavolineata*. In the two species of the Ommatolampidinae, the occurrence of AflaSAT-1 was also tested using FISH, and no signals were observed (data not shown). However, with PCR amplification, the AflaSAT-1 monomer was detected in *A. dilecta*, *V. rugulosa* and *R. bergi* and further confirmed through sequencing of PCR products for each species (Additional file 2).

### Transcriptional activity of AflaSAT-1

**Fig. 6:**
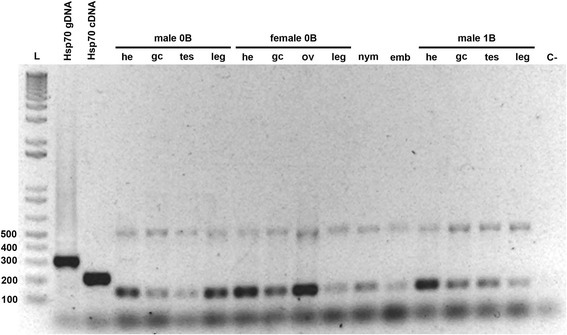
RT-PCR electrophoresis of AflaSAT-1 using as template cDNA obtained from different tissues, nymphs and embryos. he = head, gc = gastric caecum, tes = testis, ov = ovariole, leg = saltatory leg, nym = nymph, emb = embryo, C- = negative control

Based on RT-PCR analysis, AflaSAT-1 was transcriptionally active in all different organ tissues tested in male and female adults, with activity also in both embryos and nymphs. The different tissues included the head, saltatory leg, testis, ovariole and gastric caecum, in addition to embryos and first stage nymphs. These diverse samples were studied to test for constitutional transcription of AflaSAT-1, which could suggest putative function for the organism. Tissues from somatic and germ line lineages were also studied to reinforce putative constitutional function. The 1B male individuals also showed transcriptional activity for AflaSAT-1. For the analyses of all tissues and life stages, the most evident bands corresponded to monomers and tetramers of AflaSAT-1 DNA (Fig. 6), and the sequencing of monomers from randomly selected samples confirmed the specific transcriptional activity of AflaSAT-1.

## Discussion

### AflaSAT-1 organization and evolution in a chromosomes of *Abracris flavolineata*

satDNA families have been described for only 12 grasshopper species, most members of Acrididae. The representation of AflaSAT-1 was low (1.04%) in the *A. flavolineata* genome but corresponded to the fourth most abundant repetitive DNA (Milani et al., unpublished data). In grasshoppers, different satDNA repeats commonly represent less than 1% of the genome, as observed *L. migratoria* and *E. monticola* [37, 44]. However, highly abundant sequences were observed in *Schistocerca gregaria* with different satDNA families representing approximately 18% (SG1) and 12% (SG2-alfa) [45]. As expected, the general chromosomal distribution of AflaSAT-1 was coincident with pericentromeric heterochromatin, a common pattern among eukaryotes and observed extensively in grasshoppers [35, 37, 44–46]. The occurrence of another satDNA, i.e., AflaSAT-2, with the same distribution as that of AflaSAT-1 indicated a more complex structure, which was demonstrated by the two types of arrangement in the *A. flavolineata* genome. This arrangement is similar to that described in *Drosophila buzzatii* for pBuM-1 and pBuM-2 satDNA subfamilies [47]. The similar distributions of non-homologous satDNAs observed in this study have also been reported in some *Drosophila* [48], rye [49] and rodents [50].

Based on interspecies analysis, AflaSAT-1 was not specific to *A. flavolineata* and was recovered from the genomes of three other species with PCR, although dot blot analysis did not reveal positive hybridization. Although not species-specific, the copy number of AflaSAT-1 in the genomes of *A. dilecta*, *V. rugulosa* and *R. bergi* is probably low, whereas AflaSAT-1 was highly amplified in the genome of *A. flavolineata*, generating a clustered organization. Divergence among sequences among the different species was discarded as an explanation for non-positive dot blot signals, because the sequencing of PCR products revealed almost no mutations among sequences. The sharing of AflaSAT-1 among different species is consistent with the “library hypothesis” by which related species share satDNA families that can be stochastically amplified or not in the diverging genomes [51]. Considering *R. bergi*, as a member of Melanoplinae, and *A. flavolineata*, *A. dilecta* and *V. rugulosa*, as members of Ommatolampidinae and the sharing of AflaSAT-1, the origin of this satDNA likely occurred in a common ancestor before the divergence of the two subfamilies, which remits to approximately less than 73 mya, corresponding to the time of origin of Acrididae [52]. Recently, the most comprehensive comparison between the satellitomes of two other grasshopper species, i.e., *L. migratoria* (Acrididae) and *E. monticola* (Pamphagidae), showed that the life span of a satDNA library is less than approximately 100 mya, corresponding to the most recent common ancestor between the two families [37]. Our result was within this life span and showed that the divergence between satDNA libraries is variable in Acrididae, considering that the AflaSAT-1 was found in some species, depending on the subfamily, but not in others.

Low variability was observed for AflaSAT-1, and additionally, almost no distinct or population-specific profile was observed, which has also been documented for other species consistent with the concerted evolution model for homogenization of repeats at the intragenomic level [53–57]. By contrast, chromosomal variability was observed for cluster number, size, distribution and abundance of repeats, depending on the population. These data indicate that a random expansion or elimination of the satDNA may occur, generating different patterns in different populations. Variation related to satDNA mapping was also found among populations of the frog *Physalaemus cuvieri* [58] and in the grasshopper *Eyprepocnemis plorans* [59], suggesting a high rate of amplification or deletion mutational events. As expected, the population-dependent higher or lower number of chromosomal clusters for AflaSAT-1 was also consistent with the increase or decrease of AflaSAT-1 copy number, corroborating the dynamic of amplification and deletion of repeats. This change in copy number could be induced by several events, such as unequal crossing-over, slippage replication, extrachromosomal circular DNA and rolling-circle, which are common for repetitive DNAs [3, 60, 61].

### AflaSAT-1 and its relationship with the B chromosome

To date, satDNA in B chromosomes has been reported in few species of grasshoppers, such as *Eyprepocnemis plorans* [15] and *Eimugus monticola* [37], which revealed important characteristics about the origin and evolution of B chromosomes. In *A. flavolineata,* AflaSAT-1 did not disclose clues concerning the specific origin of the B chromosome because of the occurrence in almost all centromeres of A complement. However, AflaSAT-1 helped to understand the composition and molecular evolution of the B chromosome. The C-positive heterochromatin blocks are absent in the B chromosome of *A. flavolineata* [24, 42], and in the chromosomal arms, different sequences are shared with the euchromatic regions of A complement. It is considered remarkable for mobile elements and anonymous sequences to be obtained from a microdissected B chromosome [43, 61]. The heterochromatin-enriched sequences of A complement that occur in the B chromosome are restricted to a small signal in the centromeric region, as described in this study for AflaSAT-1 and for the *C*_*0*_ *t*-1 DNA [24]. Together, these data suggest that the heterochromatic sequences were not highly amplified in the B chromosome of *A. flavolineata*, as is frequently observed in other species [18], and that this chromosome most likely bears unknown euchromatic sequences. Hence, genes and other satDNAs should be investigated for a clearer picture regarding this scenario.

Molecular sequence analysis of AflaSAT-1 recovered from the microdissected B chromosome revealed exclusive mutations, with a higher number of variable sites and mutations in this chromosome. The higher sequence variability of B chromosome than that of A complement is commonly reported and has been attributed to the higher tolerance of mutations because of the dispensable nature of chromosome B [17]. Similar data are also observed for other types of repetitive DNAs, such as histone genes and 18S rRNA gene in the grasshopper *L. migratoria* [62] and in the fish *Astyanax paranae* [63]. However, in *E. plorans*, two different sequences (satDNA and 45S rDNA), and in *A. flavolineata*, the U2 snDNA, revealed different homogenization patterns with high similarity between A and B chromosomes [61, 64]. The discrepancy in sequence variability observed for the two repetitive DNAs in the B chromosome of *A. flavolineata*, i.e., AflaSAT-1 and U2 snDNA, suggests that different sequences with variable roles have divergent evolutionary patterns.

### AflaSAT-1 is transcriptionally active satellite sequence

SatDNA transcripts have been reported in different eukaryotes, and the evidence is accumulating that satellite transcription might be a common placement with putative structural or functional role for genomes [10]. Included among the important roles of satDNAs are heterochromatin formation and regulation, involvement in centromere function, epigenetic chromatin silencing and modulation, and regulation of genes, among others [65].

Among insects, satDNA transcription has been described generally in Hymenoptera, Orthoptera, Diptera and Coleoptera [66] and in the lepidopteran species *Cydia pomonella* [67] and *Plodia interpunctella* [68]. Differential transcription of satDNAs in this group is related to different developmental stages and tissues and also the sexes [54, 69, 70]. Considering this information and the constitutional transcription of AflaSAt-1, we suggest an unknown biological function for AflaSAT-1 satDNA, such as a regulatory element, with a structural or functional role, in the *A. flavolineata* genome. In future research, the quantitative differential transcription of AflaSAT-1 in *A. flavolineata*, depending on tissue, sex and presence/absence of B chromosome, must be determined to obtain more precise information for putative functional roles.

## Methods

### Animal sampling

A total of 129 adult *A. flavolineata* were collected at five different sites in Brazil (BR) and one from Argentina (AR): Rio Claro/SP (São Paulo), 22°24′45″ S, 47°31′28″ W (30 individuals); Cabo/PE (Pernambuco), 8°17′15″ S, 35°2′7″ W (19 individuals); Sta. Bárbara do Pará/PA (Pará), 1°13′27″ S, 48°17′38″ W (60 individuals); Paranaíta/MT (Mato Grosso), 9°40′25″ S, 56°28′36″ W (5 individuals); Manaus/AM (Amazonas), 3°06′02″ S, 59°58′31″ W (4 individuals); and Posadas/Misiones/AR, 27^o^25’ S, 55^o^56’ W (14 individuals). Testes were fixed in 3:1 absolute ethanol:acetic acid and stored at −20 °C. Entire animals were immersed in absolute ethanol and stored at −20 °C for genomic DNA (gDNA) extraction. We obtained tissues for DNA extraction and for chromosomes from Rio Claro/SP, Cabo/PE, Sta Bárbara do Pará/PA and Posadas/AR populations, whereas from Manaus/AM and Paranaíta/MT populations, only tissues for DNA extraction were obtained. For each analysis, at least three individuals were used.

To obtain embryos, animals collected in Rio Claro/SP were placed in plastic boxes until oviposition. Embryo mitotic cells were obtained from embryo dissection approximately 15 days after deposition, following [71] method.

### Restriction enzymatic digestion, cloning and primer design

The genomic DNA was extracted using the phenol-chloroform method [72] and stored at −20 °C until use. Genomic DNA of the specimens from the Rio Claro/SP population was fragmented by restriction enzymatic digestion using *Hind*II, *Alu*I and *Sma*I enzymes. The digested products were fractionated by electrophoresis in 1% agarose gel to verify a ladder pattern, typical for in tandem DNA sequences. Fragments with a ladder pattern were selected and purified using a Zymoclean™ Gel DNA Recovery Kit (Zymo Resarch Corp., The Epigenetics Company, USA) according to the manufacturer’s instructions.

The purified products were cloned using a pMOS*Blue* Blunt Ended Cloning Kit (GE Healthcare) with DH5α *Escherichia coli* as competent cells. Positive colonies were randomly chosen and screened by Polymerase Chain Reaction (PCR) using M13 primers set (F-5’GTAAAACGACGGCCAG and R-5’CAGGAAACAGCTATGAC) for DNA sequencing by Macrogen Inc. (Korea). Geneious v4.8.5 software [73] was used to check quality and exclude vector sequences. To amplify the satDNA sequence (AflaSAT-1) using PCR, the specific primer set (F-5’GACAGTTTTAAACACTTCCATTACAG and R-5’GACTGTGTTGATATCCAATAACA)was designated.

### PCR amplification and sequence analysis

Using specific primers for AflaSAT-1, PCR amplification was conducted using the genomic DNA as template of animals from the six different populations and from the B chromosome previously microdissected (μB-DNA) from individuals from Rio Claro/SP [61]. PCR products were visualized on a 1% agarose gel, and the bands were isolated and purified using a ZymocleanTM Gel DNA Recovery Kit (Zymo Research Corp., The Epigenetics Company, USA) according to the producer’s recommendations.

For cloning of the purified PCR products, pGEM-T easy vector (Promega, Madison, WI, USA) was used with DH5α *Escherichia coli* as competent cells. Positive clones were screened using the M13 primers and sequenced by Macrogen Inc. (Korea).

The monomer sequences were analyzed using Geneious v4.8.5 [73], and then, DNA polymorphism and haplotype recognition were checked using the DnaSP v.5.10.01 tool [74]. For more accurate analysis, singleton sequences were discarded. A graphical haplotype network was constructed employing Network 4.6.1.2 software (http://www.fluxus-engineering.com). The correspondent haplotype sequences were deposited in NCBI database under the accession numbers MF752447-MF752457.

We searched the AflaSAT-1 repeats in the sequenced genome of *A. flavolineata* from an individual harboring one B chromosome. This genome was sequenced using Illumina Miseq paired-end (2 × 300), and the libraries were constructed using a Nextera DNA Library Preparation Kit and quantified by a KAPA Library Quantification Kit (Milani et al., in preparation). For these purposes, we applied the graph-based clustering and assembly using RepeatExplorer [75] and manually searched for sequences similar to AflaSAT-1 in the assembled contigs using Geneious v4.8.5 [73]. For this search, we considered only clusters showing high graph density that are typical for satDNA [76]. In the clusters containing the AflaSAT-1, we also observed another associated sequence (named AflaSAT-2), and a primer set to recover this sequence was designed, F-5’GGGTCTCGCGAAATGAGAC and R-5’GCTTTCTAAACGGAATCGAG.

### Chromosome obtaining and fluorescent in situ hybridization (FISH)

Meiotic cells were obtained from testes, whereas mitotic cells were obtained from embryos. For conventional analyses used to check the general chromosomal structure of animals from different populations, the slides were prepared with tissue maceration and staining with Giemsa 5%.

The PCR products from satDNAs from Rio Claro/SP individuals were used as probes for FISH assays using chromosomes of individuals from the populations of Rio Claro/SP, Cabo/PE, Sta Bárbara do Pará/PA and Posadas/AR. Fragments were labeled by nick translation using digoxigenin-11-dUTP and detected by anti-digoxigenin rhodamine (Roche, Mannheim, Germany) or biotin-14-dUTP detected with streptavidin Alexa Fluor-488 conjugated (Invitrogen, San Diego, CA, USA). FISH experiments were conducted following [77], with adaptations by [78]. Fiber-FISH was conducted according to [79]. Slides were counterstained with DAPI (4′,6-Diamidine-2′-phenylindole) and mounted with VECTASHIELD (Vector, Burlingame, CA, USA). Pictures were captured using a DP70 cooled digital camera in gray scale coupled with an Olympus microscope BX51 equipped with a fluorescence lamp and appropriate filters. The images were pseudo-colored, merged and treated for brightness and contrast using Adobe Photoshop CS6.

### Dot blot hybridization

To determine whether the AflaSAT-1 was shared with other species at the level of genus, subfamily and family, we conducted a dot blot experiment, following the descriptions from [80], using the sequence amplified from individuals from Rio Claro/SP as a probe for the other six species. The probe was tested against the genomic DNA of *A. flavolineata* as positive control, the congeneric species *A. dilecta* and one another species in the same subfamily (Ommatolampidinae), i.e., *Vilerna rugulosa*. Moreover, we used species from other subfamilies of Acrididae, including Melanoplinae (*Ronderosia bergi*), Cyrtacanthacridinae (*Schistocerca pallens*), Gomphocerinae (*Amblytropidia robusta*) and Leptysminae (*Eumastusia koebelei koebelei*). The presence of AflaSAT-1 was also tested using PCR in each species following the same conditions applied for *A. flavolineata* described above, and the results were checked by nucleotide sequencing.

### Copy number detection by qPCR

To verify the copy number of AflaSAT-1 among different populations and in individuals harboring one B chromosome, we performed a relative quantification employing the gene dose ΔCt method [81]. qPCR assays were performed using the gDNA from male samples of each population for which we obtained FISH results, i.e., Cabo/PE, Sta Bárbara do Pará/PA and Posadas/AR. For the Rio Claro/SP population, we tested males with and without one B chromosome and females without a B chromosome. The gene dosage ratios (GDR) were obtained following the same parameter of [25], using a Heat Shock Protein (Hsp70) as the reference gene (F-5’GGTGTGATGACCACTCTTATCAA and R-5’CACTTCAATTTGAGGCACACC), because no difference was detected in amplification ratio among males, females and 0B and 1B individuals. Our results support that the Hsp70 gene is placed in autosomes, considering no difference between males and females. The target and reference gene were analyzed simultaneously in duplicates of four independent samples, and the qPCR conditions were set at 95 °C for 10 min; 45 cycles of 95 °C for 15 s, and 60 °C for 1 min, performed in a StepOne Real-Time PCR System (Life Technologies, Carlsbad, CA). Specificity of the PCR products was confirmed by analysis of the dissociation curve.

### RNA extraction and transcriptional analyses

The RNA was extracted from organ tissues of male and female adults, including head, saltatory leg, testis, ovariole and gastric caecum, and from embryos (with 20 days of development) and first stage nymphs (immediately after hatch) from the Rio Claro/SP population. We also tested these tissues from adult males with 1B. RNA was extracted using TRIzol® Reagent (Life Technologies) and then treated with Amplification Grade DNAse I (Sigma-Aldrich) to avoid DNA contaminants. Finally, the cDNA samples were obtained by reverse transcription (RT-PCR) using a High-Capacity cDNA Reverse Transcription Kit (Life Technologies) with the reaction conditions set to 25 °C for 10 min; 37 °C for 120 min, and 85 °C for 5 min. Afterward, the cDNA samples were used as templates for conventional PCR amplification of AflaSAT-1, with at least three biological replicates.

To validate the signals of transcription and to ensure that the samples were not contaminated with genomic DNA, the cDNA samples were used as source for amplification of Hsp70 as a control. The amplification of the Hsp70 gene in genomic DNA samples and uncontaminated cDNA samples revealed bands with different sizes of approximately 300 and 200 bp, respectively. An intron in the genomic DNA that was not present after RNA transcription and processing caused the difference. Some PCR products were sequenced to confirm the HSP70 sequence.

## Additional files

Additional file 1: qPCR data of gDNA used to calculate the sequence dose by Ct method of relative quantification (Nguyen et al. 2013). Gene dosage ratios (GDR) of the target genes were compared with autosomal gene Hsp70. (XLSX 90 kb)

Additional file 2: (a) Dot blot analysis, (b) PCR electrophoresis of AflaSAT-1 and (c) Nucleotide alignment of positively amplified AflaSAT-1 fragments. Af = *Abracris flavolineata*, 1 = *A. dilecta*, 2 = *Vilerna rugulosa*, 3 = *Ronderosia bergi*, 4 = *Schistocerca pallens*, 5 = *Amblytropidia robusta*, 6 = *Eumastusia koebelei koebelei*. (JPEG 363 kb)

## Abbreviations

2n: Diploid number
Bp: Base pairs
DAPI: 4′, 6-Diamidino-2-phenylindole
FISH: Fluorescence in situ hybridization
PCR: Polymerase chain reaction
satDNA: Satellite DNA
