# Supplementary figures and images for: The satellite DNA AflaSAT-1 in the A and B chromosomes of the grasshopper Abracris flavolineata
Source: BMC Genet. 2017 Aug 29;18:81. doi: 10.1186/s12863-017-0548-9 (PMC5575873; doi:10.1186/s12863-017-0548-9)

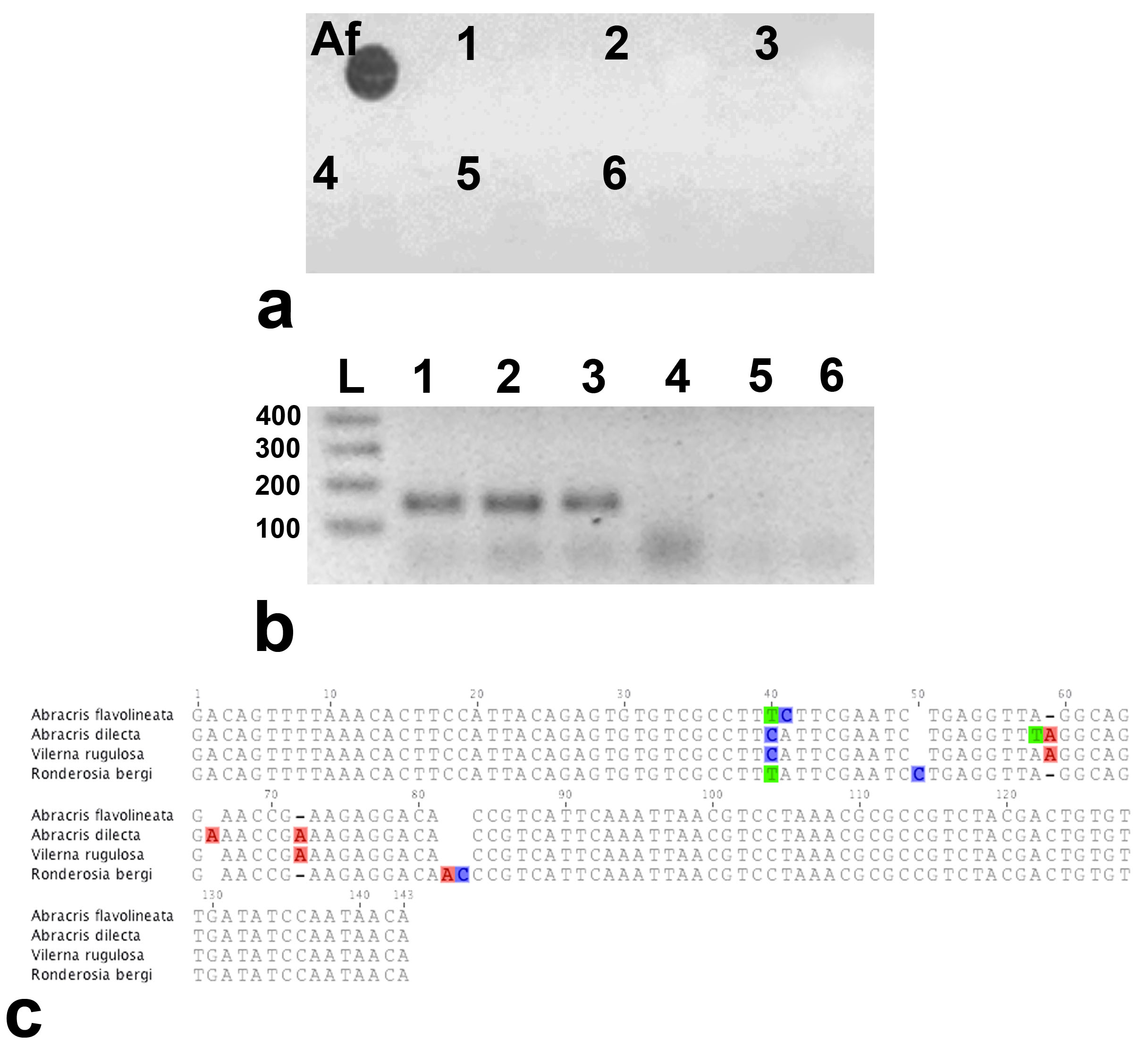

Supplement: Supplementary file 2 — (a) Dot blot analysis, (b) PCR electrophoresis of AflaSAT-1 and (c) Nucleotide alignment of positively amplified AflaSAT-1 fragments. Af = Abracris flavolineata, 1 = A. dilecta, 2 = Vilerna rugulosa, 3 = Ronderosia bergi, 4 = Schistocerca pallens, 5 = Amblytropidia robusta, 6 = Eumastusia koebelei koebelei. (JPEG 363 kb) [file 12863_2017_548_MOESM2_ESM.jpg]
